# Electroacupuncture Ameliorates Acute Renal Injury in Lipopolysaccharide-Stimulated Rabbits via Induction of HO-1 through the PI3K/Akt/Nrf2 Pathways

**DOI:** 10.1371/journal.pone.0141622

**Published:** 2015-11-02

**Authors:** Jian-bo Yu, Jia Shi, Yuan Zhang, Li-rong Gong, Shu-an Dong, Xin-shun Cao, Li-li Wu, Li-na Wu

**Affiliations:** Department of Anesthesiology, Tianjin Nankai Hospital, Tianjin Medical University, Tianjin, China; Center for Molecular Biotechnology, ITALY

## Abstract

Electroacupuncture at select acupoints have been verified to protect against organ dysfunctions during endotoxic shock. And, heme oxygenase (HO)-1 as a phase II enzyme and antioxidant contributed to the protection of kidney in septic shock rats. The phosphatidylinositol 3-kinase (PI3K)-Akt pathway mediated the activation of NF-E2 related factor-2 (Nrf2), which was involved in HO-1 induction. To understand the efficacy of electroacupuncture stimulation in ameliorating acute kidney injury (AKI) through the PI3K/Akt/Nrf2 pathway and subsequent HO-1 upregulation, a dose of LPS 5mg/kg was administered intravenously to replicate the rabbit model of AKI induced by endotoxic shock. Electroacupuncture pretreatment was handled bilaterally at Zusanli and Neiguan acupoints for five consecutive days while sham electroacupuncture at non-acupoints as control. Results displayed that electroacupuncture stimulation significantly alleviated the morphologic renal damage, attenuated renal tubular apoptosis, suppressed the elevated biochemical indicators of AKI caused by LPS, enhanced the expressions of phospho-Akt, HO-1protein, Nrf2 total and nucleoprotein, and highlighted the proportions of Nrf2 nucleoprotein as a parallel. Furthermore, partial protective effects of elecroacupuncture were counteracted by preconditioning with wortmannin (the selective PI3K inhibitor), indicating a direct involvement of PI3K/Akt pathway. Inconsistently, wortmannin pretreatment made little difference to the expressions of HO-1, Nrf2 nucleoprotein and total protein, which indicated that PI3K/Akt may be not the only pathway responsible for electroacupuncture-afforded protection against LPS-induced AKI. These findings provide new insights into the potential future clinical applications of electroacupuncture for AKI induced by endotoxic shock instead of traditional remedies.

## Introduction

Septic shock is the most common contributing factor to acute kidney injury (AKI) in critically patients, and even progress into acute renal failure [1]. The combination of acute renal failure and sepsis accounts for more than 70% mortality, despite multifarious potential interventions [2]. Lipopolysacchride (LPS) is a primary initiator of inflammatory and hemodynamic perturbations of sepsis and involved in experimental AKI studies [3]. There is experimental evidence that endotoxin-related increase in tumor necrosis factor-α (TNF-α) and oxygen radicals are major causes of AKI during endotoxic shock [4, 5]. Heme oxygenase (HO)-1, as the rate limiting enzyme of heme degradation, was first recognized in preserving renal function and reducing mortality [6]. Our prior studies definitely elucidated the protective role of HO-1 on the kidney in endotoxic shock rats [7]. The biological effect of HO-1 attributed to the degradation of heme and the products of enzymatic reaction including biliverdin and carbon monoxide, which exerted antioxidant, antiproliferative and also anti-inflammatory properties [8].

Acupuncture represents a potentially valuable adjunct to prevent clinical disorders in China and other Asian countries for the past 3000 years [9]. It was proven that electrical stimulation of the vagus nerve could modulate systemic inflammatory responses to endotoxin, inhibit the synthesis of TNF and prevent the development of shock [10]. In addition, electroacupuncture was found to produce neuro-protective effect by enhancing the activities of antioxidant enzymes and reducing the extent of lipid peroxidation [11]. Traditionally, Zusanli (ST36) and Neiguan (PC6), two specific points, were considered of choice for good health care. Recent studies have shown that ST36 acupuncture pretreatment exerted protective effects against sepsis-induced kidney injury [12]. Besides, Liu et al indicated that electroacupuncture at PC6 retrieved blood pressure and improved endotoxic shock in rats [13]. However, little was known about its mechanisms of modulation.

Several transcriptional factors including NF-E2 related factor-2(Nrf2), activator protein (AP)-1 and nuclear factor-κβ (NF-κβ) have been implicated in the regulation of HO-1 expression [14]. Among these, Nrf2 played a crucial role in mounting the innate immune response and maintaining redox homeostasis, which determined survival during endotoxic shock [15]. Moreover, only phosphatidylinositol 3-kinase (PI3K)-related signal pathway controlled the activation of Nrf2 was involved in the induction of HO-1 [16, 17]. Meanwhile, PI3K/Akt pathway was thought to be pivotal in the maintenance of homeostasis and the integrity of the immune response during sepsis [18]. Our preliminary studies have confirmed that

electroacupuncture exerted anti-inflammatory or antioxidant capacity to reverse lung injury induced by endotoxic shock through upregulation of HO-1 and activation of Nrf2/ARE pathway [19]. Nevertheless, the role of HO-1 in protective effect of electroacupuncture against endotoxic shock induced AKI and the regulatory mechnisms have been unconfirmed systematically.

Based on these previous data, we hypothesized that electroacupuncture stimulation at bilateral ST36 and PC6 acupoints could attenuate AKI evoked by endotoxic shock in rabbits via the induction of HO-1 through modulating PI3K/Akt/Nrf2 pathway.

## Materials and Methods

### Animal preparation

For the current study, two-month-old male New England white rabbits weighing 1.5–2.0kg were obtained from Laboratory Animal Center of Nankai Clinical Institution of Tianjin Medical University. All animal experiments were carried out in strict accordance with the recommendations in the Guide for the Care and Use of Laboratory Animals of the National Institutes of Health. The protocols were approved by the Committee on the Ethics of Animal Experiments of Tianjin Nankai Hospital (NKH-20131107). The rabbits were housed at 18~22°C, and acclimatized to a 12/12-h light-dark regimen. Food and water were supplied *ad libitum* for a 5-day period before the experiment procedures. The night before surgery rabbits were denied access to food but had access to water. All operations were performed under the anesthesia of urethane, and all efforts were made to minimize the suffering of the animals.

### Electroacupuncture protocols

Two pairs of stainless steel needles (diameter 0.3mm) were inserted perpendicularly to a depth of 5mm and then held in place. The selected acupoints were Zusanli(ST36), located between the tibia and fibular approximately 5 mm lateral to the anterior tubercle of the tibia, and Neiguan (PC6), located between the radius and ulna probably one-sixth the length of the line from the wrist to elbow [12,20]. A series of nonacupoints located 5mm lateral to ST36 or PC6 original location served as controls. As described in detail previously [19, 21], stimulation (alternating disperse-dense wave of 2Hz and 15Hz, once a day) were delivered using an electrical stimulation device (HANS G6805-1A, Huayi Co, Shanghai, China) for 15 minutes. Current intensity (≤1.0mA) was adjusted to elicit slight twitches of the limbs. Electroacupuncture was initiated for a five-consecutive-day before the experiment and throughout the operating steps for 6h during the experimental day [22]. For acupuncture sessions, the rabbits were lightly immobilized using hands to minimize stress. Acupuncture points were identified by an experienced acupuncturist (ZY), who was not involved in data analysis.

### Experimental design

Rabbits were sedated with 5mg/kg of 20% urethane via the marginal ear vein. Anesthesia was maintained with continuous infusion of ketamine at 3mg/kg/h throughout the experiment. Briefly, tracheotomy was performed aseptically so as to facilitate spontaneous respiration. The animals were placed on a heating pad under a radiant heat lamp to keep the body temperature at 37.7–40.3°C. A PE-50 catheter was inserted through the right internal carotid artery to monitor arterial pressure continuously by using Hellige monitor instrument (Germany). The internal jugular vein was cannulated with a 24-g catheter for drugs administration. A retention catheter was inserted into the bladder via urethra for urine collection. Lactated Ringer’s solution (8ml/kg/h) was infused intravenously for the duration of each experiment.

**Fig 1:**
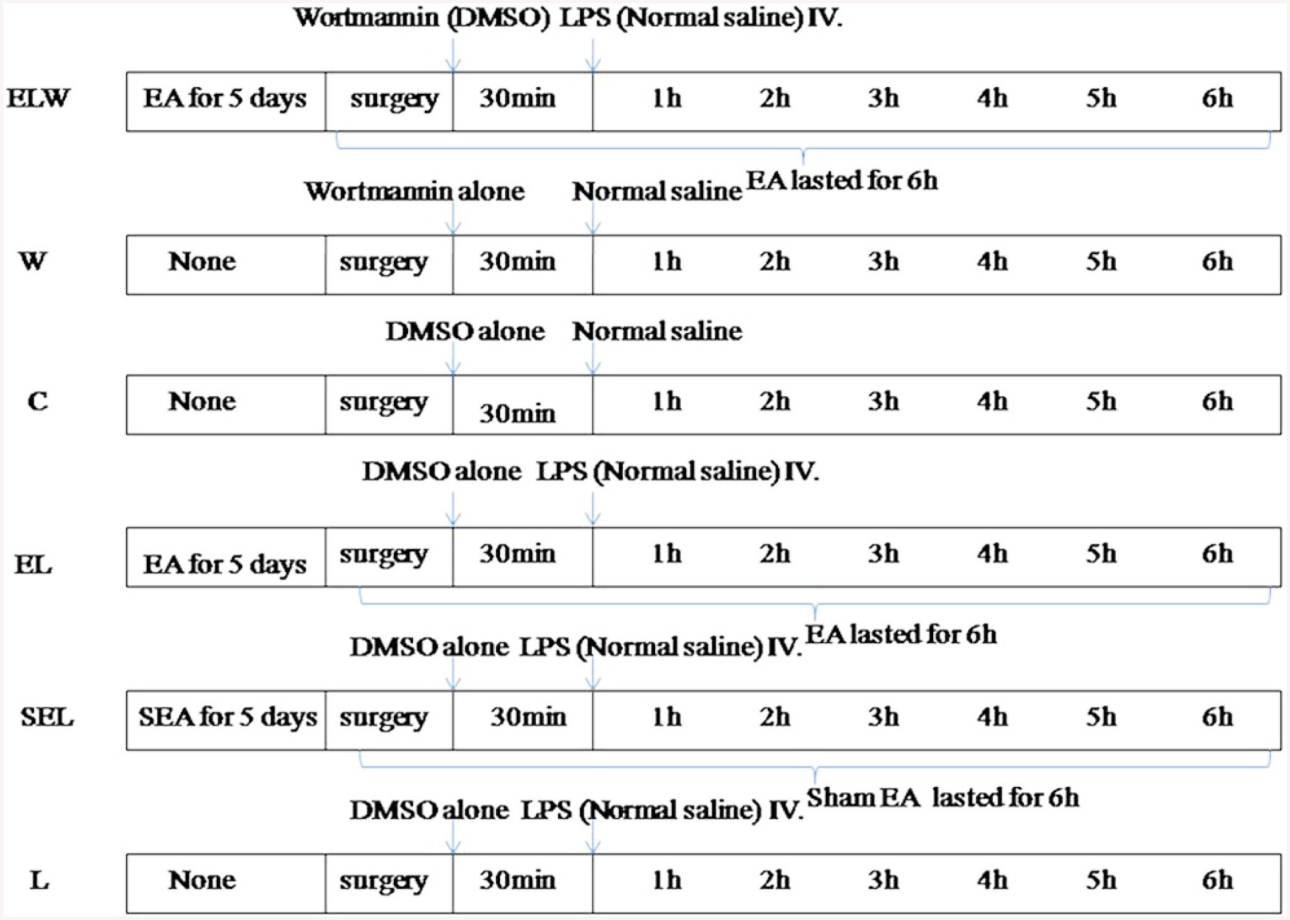
Schematic diagram of depicting the experimental protocols. “EA” presented electroacupuncture at bilaterally Zusanli and Neiguan acupoints; “SEA” presented sham electroacupuncture at non-acupoints; “none” means no electroacupuncture pretreatment for five days. “surgery” means the experimental operation of rabbits. “. “Brace” means electroacupuncture or sham electroacupuncture throughout the operation of rabbits.

Sixty rabbits were randomly assigned to six groups (n = 10/group): group ELW, W, C, EL, SEL, and L (Fig 1). Rabbits subjected to 0.5ml (5mg/kg) LPS (L2630, sigma, USA) in group ELW, EL, SEL and L to mimic endotoxic shock-induced acute kidney injury [23]. To serve as controls, the rabbits in group C received an equal amount of normal saline. Electroacupuncture treatment was applied bilaterally at ST36 and PC6 points in group EL and ELW, while group SEL acupunctured at non-acupoints as described above. To block PI3K/Akt signaling pathway, wortmannin (the selective PI3K inhibitor, 0.6mg/kg; Selleck, USA) was administered in group ELW intravenously 30min before LPS injection [24]. Meanwhile, its drug vehicle, dimethyl sulfoxide (DMSO, 0.08ml/kg) was given in other groups instead, and W group rabbits were accepted wortmannin alone. Mean arterial pressure (MAP) was monitored consecutively after LPS administration. MAP did not decrease within 2h or rabbits died within 6h were deemed as the exclusion criteria of current study [7].

### Sampling and storage

The overall health status of all rabbits enrolled in current study were monitored every 30 min~1h for indicators of distress following the injection of LPS. In particular, as with the suggestions of Nemzek et al, rabbits were euthanized by CO2 asphyxiation either at the end of the experiment, namely at 6h after LPS administration, or upon signs of imminent death including inability to ambulate, labored breathing and cyanosis, prolonged hypothermia, and unconsciousness to external stimuli [25, 26]. The blood sample was drawn from the right internal carotid artery into EDTA tubes immediately. Then, the blood specimen was centrifuged at 3000 rpm for 15min at 4°C and the supernatant was frozen at -80°C for subsequent analysis. In addition, urine samples from the bladder were extracted. After the rabbits were euthanized, the kidneys were harvested immediately and quickly perfused with phosphate-buffered saline (PBS) to remove blood. The left kidney tissues were snap frozen in liquid nitrogen and stored at -80°C for later analyses. Portions of the remnant kidney tissues were fixed in 4% formaldehyde for histopathological analysis.

### Biochemical measurements

Superoxide dismutase (SOD) activities and malondialdehyde (MDA) contents in the renal tissues were respectively quantified by spectrophotometry [27] and by means of Loewenberg [28], both of which were expressed as per unit of protein determined by the Lowry method [29]. Besides, Plasma levels of blood urea nitrogen (BUN) and creatinine (Cr) were evaluated by a Hitachi 7060 Fully Automated Biochemistry Analyzer (Tokyo, Japan). Urine concentrations of N-acetyl-glucosaminidase (NAG) were determined by para nitrophenol colorimetry (Kit supplied by Shanghai Debo Biotechnology limited company, Shanghai, China). In addition, tumor necrosis factor-alpha (TNF-a) and interleukin-10 (IL-10) levels in plasma were measured with commercially available enzyme-linked immunosorbent assay kits (R&D systems, Minneapolis, MN, USA). All the procedures were performed according to manufacturer protocols.

### Histopathological examination

Briefly, formalin-fixed paraffin-embedded kidney were sectioned at 4μm and stained with hematoxylin and eosin for general histological assessment. The semiquantitative analysis of the kidney slides were conducted by a trained histopathologist with no knowledge of treatment allocation. Each successive field was assessed individually for severity of tubulointerstitial lesions and graded as follows [30, 31]: 0, normal; 1, areas of tubular epithelial cell swelling, vacuolar degeneration, necrosis and desquamation involving <25% of cortical tubules; 2, similar changes involving >25% but <50% of cortical tubules; 3, similar changes involving >50% but<75% of cortical tubules; 4, similar changes involving>75% of cortical tubules. At least 10 different fields of each slice were visualized by microscopy (×400). The mean scores of all fields were taken as renal injury scores of above kidney sections

### TUNEL staining

Paraffin-embedded renal sections (4μm) were stained with the terminal deoxynucleotidyl transferase mediated deoxyuridin triphosphate nick-end labeling (TUNEL) assay using an in situ apoptosis detection kit (Roche, Germany) according to the manufacturer’s instructions. Brown labeled TUNEL positive cells were counted in ten randomly selected fields from each slides at a magnification of ×200, and three different kidney sections were observed for per animals (n = 10/group) by light microscopy. The TUNEL-positive cells for each field were quantified as the percentage of total renal cells counted by a blind observer [21].

### Western blot analysis

Tissue samples were homogenized in lysis buffer solution (13.2 mmol/L Tris-HCl, 5.5% glycerol, 0.44% SDS and 10% β-mercaptoethanol). After the supernatants were collected, the protein extracts were obtained by using the total and nuclear protein isolation kit (Thermo, USA). Protein levels were detected by the Bicichoninic acid (BCA) assay kit (Thermo, USA). Equal amounts of separated protein (50μg) were fractionated by 12% SDS-PAGE and transferred to a PVDF membrane (Bio-Rad, USA). Blots were incubated overnight with at 4°C with polyclonal rabbit antibodies against phosphor(Ser 473)-specific Akt (1:300, Bioss Inc, USA), HO-1(1:800, Abcam, UK) and Nrf2 (1:300, Biorbyt, UK). Then, blots were washed triple for 10 min with TBS-0.05% Tween 20, and incubated with 1:3000 dilution of the horseradish peroxidase (HRP)-conjugated goat anti-rabbit IgG (Biorbyt, UK) for 1h at 37°C. Antigenic detection was visualized by enhanced chemiluminesence (Bio-Rad, USA) as described by Wang et al [5], and the relative density of bands was quantified by densitometry (Molecular Analyst Image-analysis Software, Bio-Rad, USA). The densitometer values of measured proteins were normalized to β-actin used as loading controls.

### Immunoflurescence assays

To estimate the intracellular distribution of Nrf2 nucleoprotein, immunoflurescence analysis was perfomed as previously described [19]. Kidney tissues were embedded in paraffin, sectioned at 4μm, and then dewaxed in xylene and rehydrated with graded ethanol solutions. After protein blockade, the sections were incubated overnight at 4°Cwith the polyclonal Nrf2 primary antibody conjugated to FITC (dilution 1:150, Biorbyt, UK). Following washing triple in phosphate-buffered saline (PBS), slides were counterstained with 4’, 6-diamidino-2-phenylindole (DAPI) (Roche, Shanghai, China) to visualize the nuclei. The images were captured by a fluorescent microscope (Olympus U-25ND25, Tokyo, Japan) coupled to a digitial camera. The overlay color of blue staining in nucleus, accompanied with green staining both in cytoplasm and nucleus was considered to be positive. A semiquantitative numeric scores were determined for kidney samples based on the percentage of positive protein in five fields of each slices at a 400 multiple signal magnification using Image-pro plus software (Image pro plus 6.0, media Cybernetics, USA) [32, 33].

### Statistical analysis

Data are expressed as means ± SD. For signal comparisons, renal injury scores were analyzed by the non-parametric unpaired Mann-Whitney test. Besides, parametric data were analyzed for statistical significance by means of one-way ANOVA, followed by the Bonferroni-Dunn method for multiple comparisons. The analyses were carried out with GraphPad Prism 5.0 (GraphPad software, La Jolla, CA, USA). *P* values < 0.05 were considered to indicate statistical significance.

## Results

### Renal SOD activities and MDA contents

**Fig 2:**
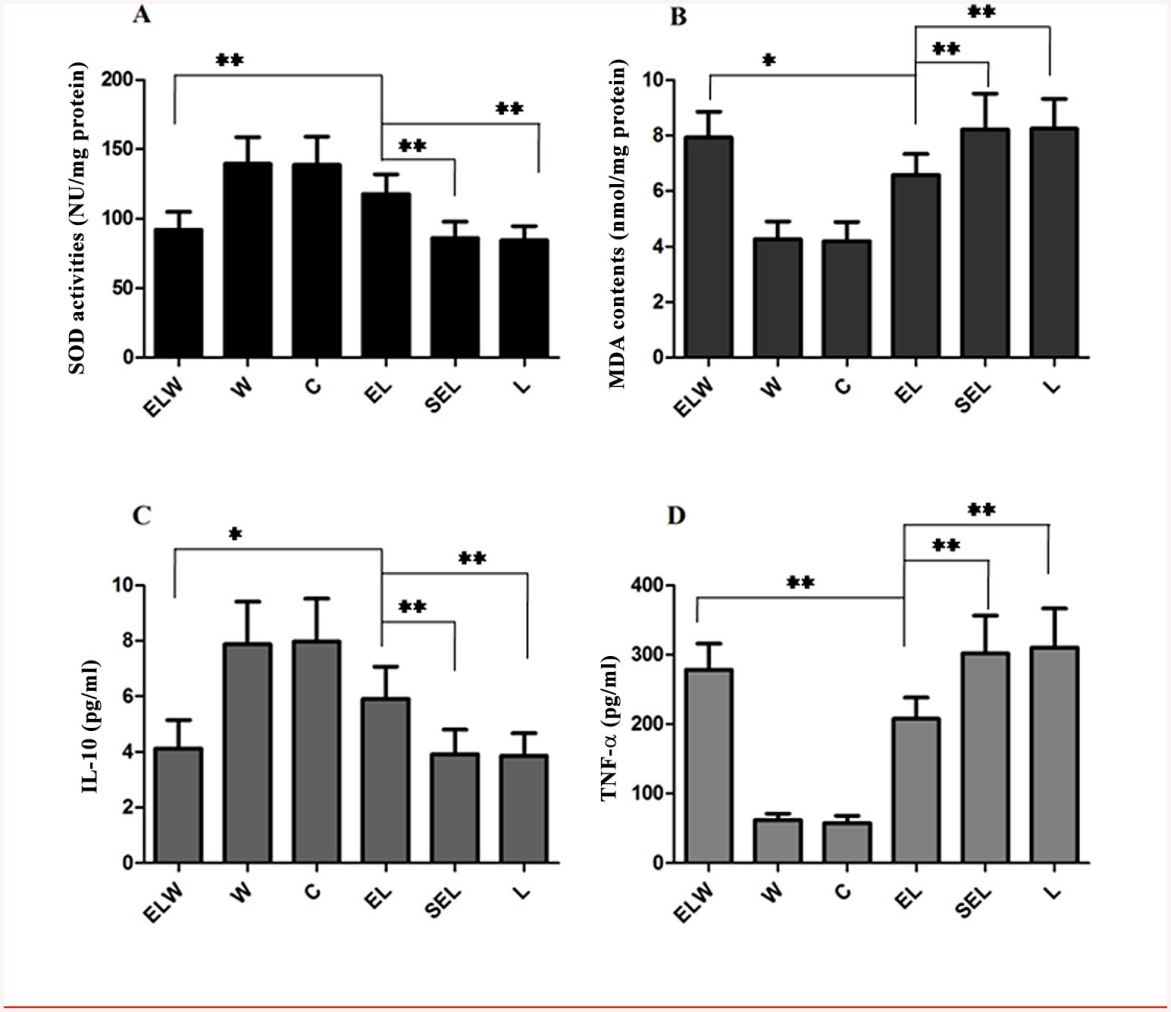
Analysis of SOD activities (A), MDA contents (B) in the renal tissue, as well as plasma IL-10 (C) and TNF-α levels (D) in each groups. Electroacupuncture at bilaterally Zusanli and Neiguan acupoints significantly increased SOD activities, decreased MDA contents accompanied with increased levels of IL-10 and decreased TNF-α levels. Pretreatment with wortmannin significantly restrained the above effects of electroacupuncture in group ELW. Values were presented as mean ± SD (n = 10). The data were analyzed using one-way ANOVA and the Bonferroni test for multiple comparisons. Significant differences were indicated with an asterisk: *P<0.*05*, **P<0.*01*.

As shown in Fig 2, LPS potently decreased the activities of SOD, while increased the contents of MDA compared to group C (all P<0.01). By contrast, electroacupuncture treatment significantly counteracted the alterations induced by LPS (P<0.01) (augment 25.4% for SOD, attenuation 20.2% for MDA). No significant influences in above parameters were discovered when compared group SEL with group L (P>0.05). Additionally, administration of wortmannin in group ELW could reverse the protective effects of electroacupuncture at ST36 and PC6 (P<0.05).

### Plasma TNF-a and IL-10

Plasma levels of TNF-α and IL-10 at 6h after LPS injection were shown in Fig 2. Electroacupuncture stimulation obviously attenuated TNF-a by 29.9% and enhanced IL-10 by 32.6% in group EL compared with group L or SEL (P<0.01). However, above protective effects were neutralized to some extent by the selective PI3K inhibitor, wortmannin (P<0.05). There were no significant differences between group L and group SEL (P>0.05).

### Plasma BUN, Cr and urine NAG

Data in Table 1 revealed that rabbits from group L, EL, SEL and ELW possessed higher levels of BUN, Cr and NAG than group C or W (P<0.01). Besides, as markers of renal function, both BUN and Cr, as well as NAG were distinctly reduced in electroacupuncture treatment group compared with group L (P<0.01). Wortmannin pretreatment notably restrained the levels of these indicators in group ELW (P<0.05). We did not find a significant difference in the levels of BUN, Cr and NAG between group SEL and group L (P>0.05).

**Table 1:** Comparison of plasma BUN and Cr, as well as urine NAG levels in the study groups.

Groups	BUN (mmol/L)	Cr (μmol/L)	NAG (U/L)
**ELW**	9.91±1.35* ^#^	108.9±16.83* ^#^	39.8±9.55* ^#^
**W**	5.64±0.95	36.1±3.48	13.2±5.16
**C**	5.68±0.87	38.4±3.56	13.7±5.23
**EL**	7.78±1.12* ^+^	82.6±13.85* ^+^	26.9±7.49* ^+^
**SEL**	10.91±1.54* ^#^	118.1±21.87* ^#^	46.5±13.72* ^#^
**L**	10.83±1.67* ^#^	120.7±23.40* ^#^	43.3±11.23* ^#^

*Abbreviations*: *BUN*, blood urea nitrogen; *Cr*, creatinine; *NAG*, N-acetyl-glucosaminidase. Values were expressed as mean ± SD (n = 10; one-way ANOVA followed by Bonferroni post-hoc test was used for statistic analysis of the data;

*P<0.*01* versus group C;

^*+*^ *P<0*.*01* versus group L;

^*#*^ *P<0*.*05* versus group EL).

### Renal histopathology

**Fig 3:**
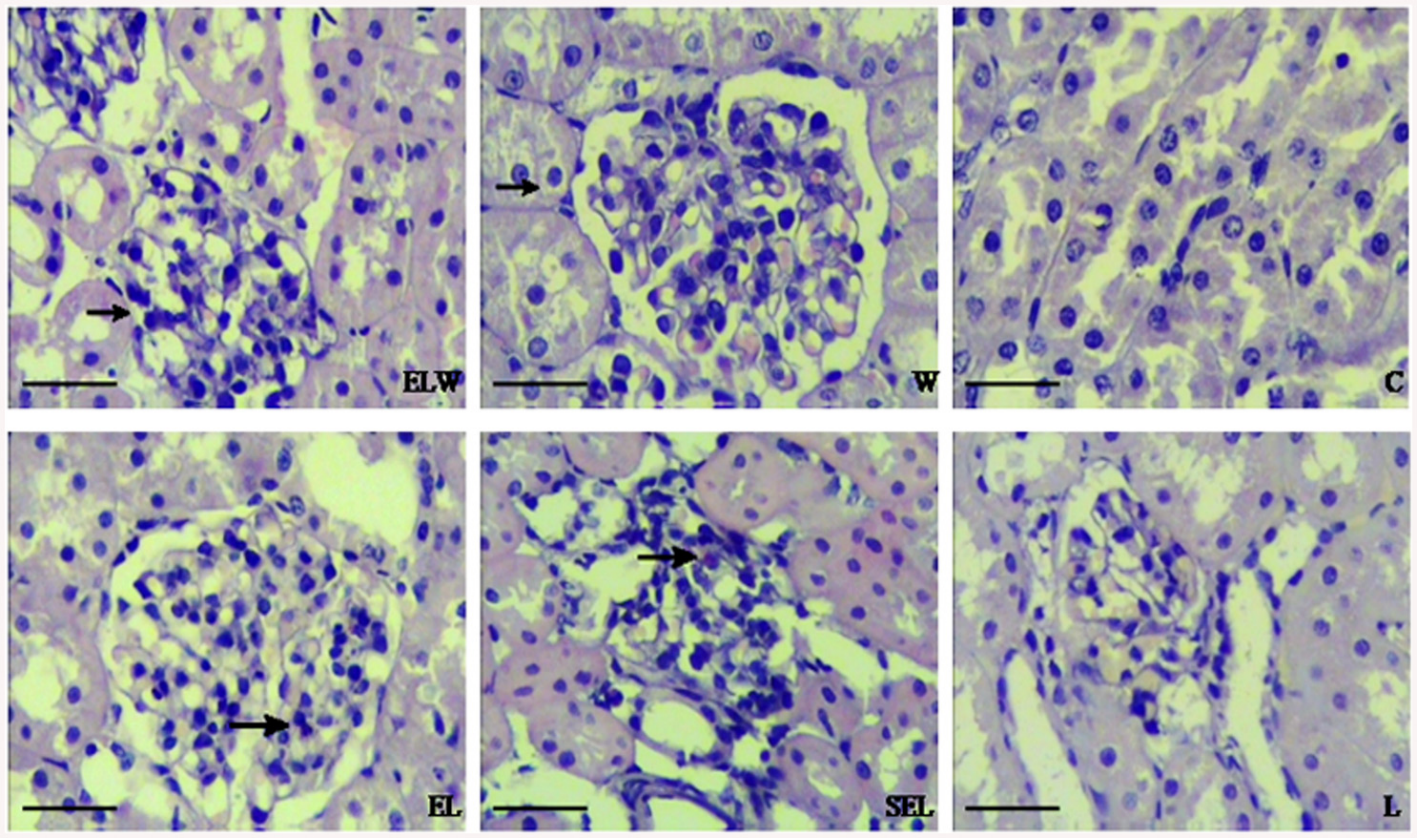
Microphotographs of histopathologic changes of kidney sections stained with hematoxylin and eosin. (original magnification×400): (W, C) Normal morphology of kidneys; (L, SEL) Rabbits treated with LPS or sham electroacupuncture showing infiltration of inflammatory cells, tubular epithelial cell vacuolization, swelling and desquamation. (EL) Marked attenuation of tubular damages were displayed in treatment with electroacupuncture at ST36 and PC6 acupoints. (ELW) Preprocessing with wortmannin suppressed the protective efficacy of electroacupuncture to some extent. Black arrows: the destruction of renal capsule and capsular space in group ELW while infiltration of inflammatory cells in group EL or group SEL.

As shown in Fig 3, no abnormal structures were visible in group C and group W. In comparison, kidney tissues from LPS group had extensive cellular vacuolization, swelling and desquamation in proximal tubules, interstitial edema and neutrophil infiltration. There was a distinct reduction in the extent of morphological damage in rabbits treated with electroacupuncture at ST36 and PC6. However, preprocessing with wortmannin suppressed the efficacy of electroacupuncture treatment. The results, presented as the scores of acute kidney injury, were summarized in Table 2. Consistently, the levels of kidney injury scores in group EL were lower than group L, and higher than group ELW (P<0.05). Meanwhile, similar results were observed in group SEL and group L (P>0.05).

**Table 2:** Semi-quantitative analysis of renal injury scores among the following six groups.

Groups	Renal Injury Scores
**ELW**	2.5±1.08* ^#^
**W**	0.4±0.52
**C**	0.5±0.53
**EL**	1.4±0.97* ^+^
**SEL**	2.9±1.20* ^#^
**L**	2.8±1.23* ^#^

Values were analyzed by the non-parametric unpaired Mann-Whitney test. (n = 10;

*P<0.*05* versus group C;

^*+*^ *P<0*.*05* versus group L;

^*#*^ *P<0*.*05* versus group EL).

### TUNEL assay

**Fig 4:**
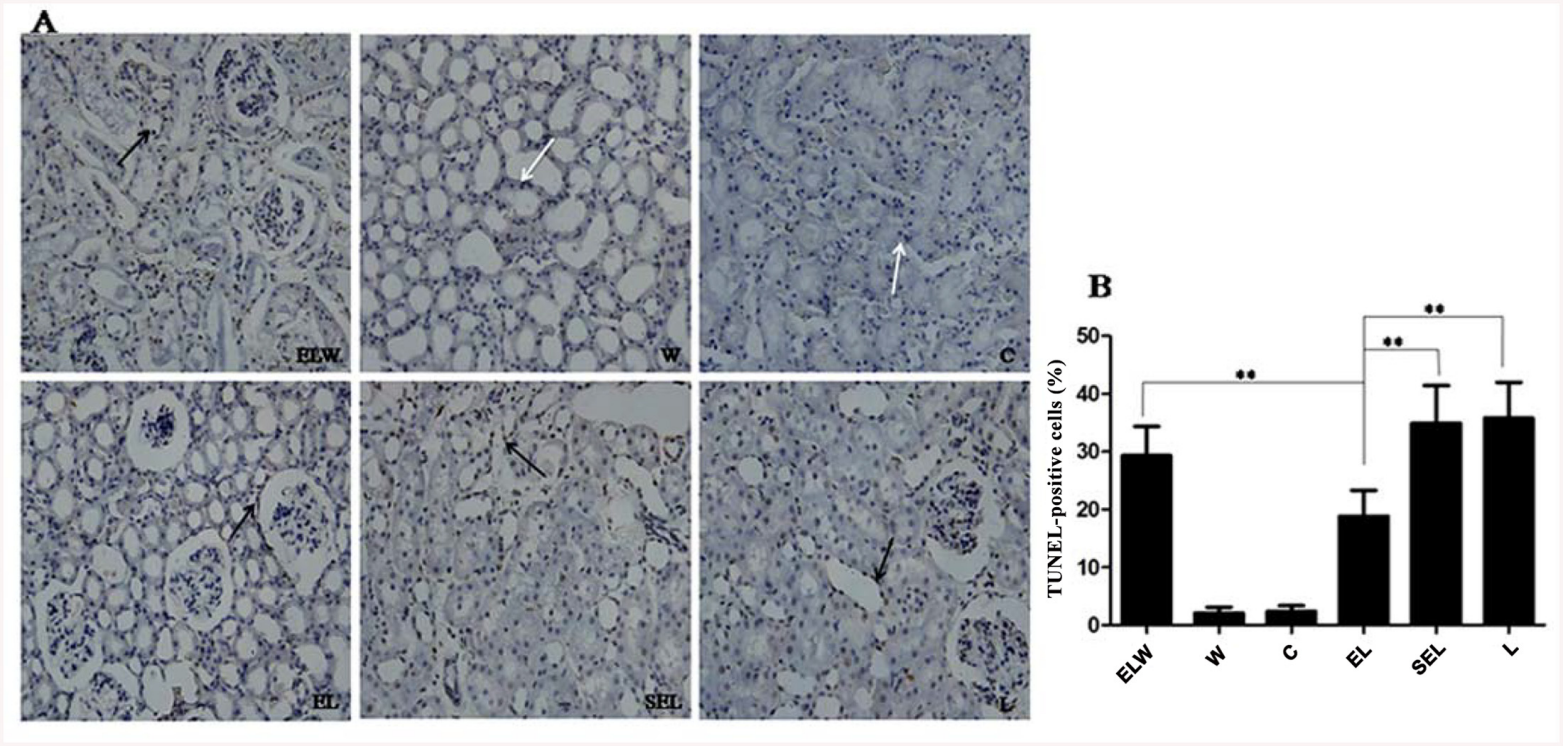
TUNEL in situ assay of tubular cell death in the renal tissues. (original magnification×200): (A) Representative photomicrographs of TUNEL stained rabbit kidneys corresponding to group ELW, W, C, EL, SEL and group L. White arrows: normal cells; Black arrows: the TUNEL-positive renal tubular cells. (B) Quantification of the number of TUNEL-positive cells using semi-quantitative analysis of the positive staining from ten randomly selected fields. The data were expressed as mean ± SD (n = 10; **P<0.*01*, using one-way ANOVA and the Bonferroni test for multiple comparisons).

To assess whether electroacupuncture stimulation decreased the amount of renal tubular cell death in LPS-induced AKI, and the proportion of apoptotic cells in renal slides were determined by TUNEL staining (Fig 4). An elevated number of TUNEL-positive tubular cells were observed in rabbits subjected to LPS than that of controls (P<0.05). In contrast to group L, electroacupuncture preconditioning caused a significant decrease in the number of TUNEL-positive cells in group EL, while wortmannin pretreatment in group ELW blocked the protective effect of electroacupuncture stimulation (P<0.05). Additionally, the percentage of TUNEL-positive cells in group SEL resembled that of group L (P>0.05).

### Renal phospho-Akt protein, HO-1 protein, Nrf2 total and nucleoprotein expressions

**Fig 5:**
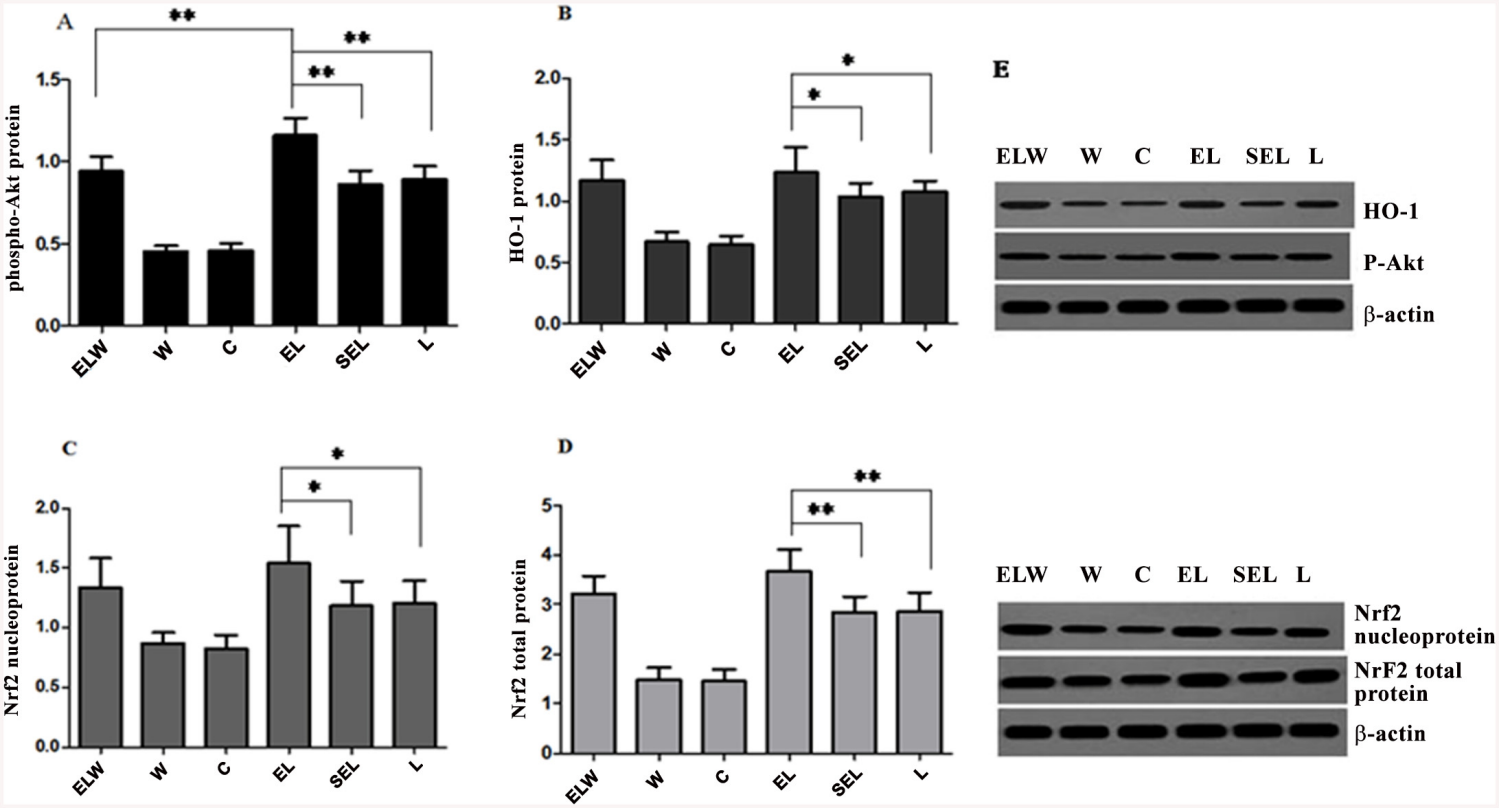
Western blot analysis of phospho-Akt protein (A), HO-1 protein (B), Nrf2 total (C) and nucleoprotein (D) expressions in the renal tissue of six groups. β-actin was monitored as the internal standard to ensure similar gel loading of the starting materials in each sample. Blot images were cropped for comparison (E). Electroacupuncture treatment significantly increased the levels of phospho-Akt protein, HO-1 protein, Nrf2 total and nucleoprotein compared with group L or group SEL. In wortmannin-treated rabbits, phosphorylation of Akt was significantly decreased while HO-1 protein, Nrf2 total and nucleoprotein was slightly decreased compared with group EL. All values were expressed as mean ±SD (n = 10; *P<0.05; **P<0.*01*, using one-way ANOVA and the Bonferroni test for multiple comparisons). The blots were representative of three independent experiments.

Exposure to LPS notably increased the expressions of p-Akt protein, HO-1 protein, Nrf2 total and nucleoprotein compared with group C (P<0.05), illustrated in Fig 5. Besides, electroacupuncture treatment markedly upregulated the levels of above mentioned proteins in contrast to group L or group SEL (P<0.05). In wortmannin-treated rabbits, phosphorylation of Akt was substantially decreased (P<0.05), while HO-1 protein, Nrf2 total and nucleoprotein were slightly decreased compared with that in group EL (P>0.05). However, there were no significant differences between group SEL and group L in terms of the measured protein expressions (P>0.05).

### Renal distribution ratio of Nrf2 nucleoprotein expression

**Fig 6:**
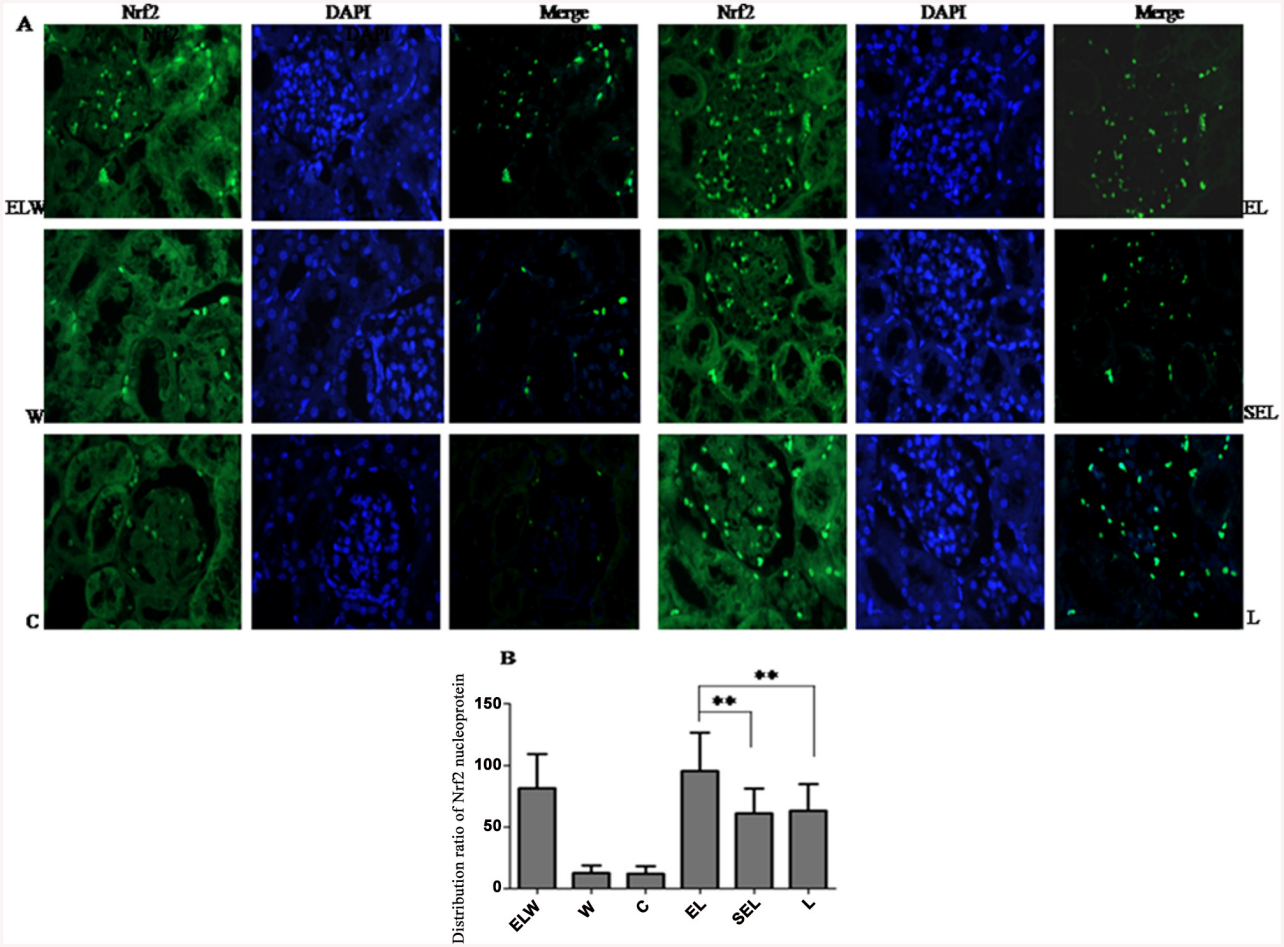
Immunofluorescence assays of Nrf2 nucleoprotein by fluorescence microscope. (original magnification×400): (A) The pictures of immunofluorescence staining. Green stands for Nrf2-FITC stained sections, while blue stands for images of DAPI stained nuclei. The overlay color of blue staining in nucleus, accompanied with green staining both in cytoplasm and nucleus was considered to be positive. (B) Quantification of nuclear localization of Nrf2 protein among six groups. Electroacupuncture protocols dramatically increased translocation of Nrf2 from cytoplasm into nucleus compared with group L or SEL. To some degree, pretreatment with wortmannin counteracted nuclear accumulation of Nrf2 protein, while wortmannin alone had no effects. Data were representative of three independent experiments. Values were expressed as mean ± SD (n = 10; **P<0.*01*, using one-way ANOVA and the Bonferroni test for multiple comparisons).

The translocation of Nrf2 from cytoplasm to nucleus by electroacupuncture could be visualized by Immunofluorescence analysis (Fig 6). After 6h of LPS administration, nuclear localization of Nrf2 was apparent compared to the negligible distribution in sham operation group (P<0.01). In addition, electroacupuncture treatment at ST36 and PC6 acupoints dramatically increased the nuclear accumulation of Nrf2 protein contrast with group L or group SEL (P<0.01). Moreover, Wortmannin was scarcely effective at inhibiting electroacupuncture-induced nuclear translocation of Nrf2, yet wortmannin alone had no effect. Sham electroacupuncture group exhibited indistinguishable expression of Nrf2 nucleoprotein compared with group L (P>0.05).

## Discussion

Data from current study revealed that electroacupuncture stimulation at bilateral ST36 and PC6 acupoints dramatically alleviated acute kidney injury during endotoxic shock in rabbits, coincident with the expressions of HO-1 protein, Nrf2 total and nucleoprotein as well as phospho-Akt protein. Furthermore, electroacupuncture treatment significantly inhibited LPS-induced tubular cell apoptosis, increased SOD activities, decreased MDA contents in renal tissue, accompanied with increased plasma levels of IL-10 and decreased plasma TNF-α to exert its anti-oxidative and anti-inflammatory efficacy. However, blockade of PI3K/Akt pathway by the administration of wortmannin subtracted the above-mentioned results in electroacupuncture treated animals. In summary, the protective effects of electroacupuncture at ST36 and PC6 were mainly confirmed against renal injury in lipopolysaccharide-stimulated rabbits via induction of HO-1 through the upstream PI3K/Akt/Nrf2 pathway.

Acute renal injury in endotoxemia patients is an independent predictor of mortality [34]. Despite recent advances in renal replacement therapy, the mortality of patients sustained sepsis-induced kidney injury remained unchanged [35]. However, the etiology of AKI during septic shock may be multifactorial. The interplay of inflammation and oxidative stress was put forward to explain the clinical phenotype of endotoxic shock-induced AKI, reflected by the higher levels of BUN and Cr in plasma [36]. In present study, the scores of acute renal injury, the number of TUNEL-positive cells, as well as plasma levels of TNF-α and renal contents of MDA were significantly increased in LPS induced groups. Meanwhile, urinary NAG, as a sensitive marker of renal tubular damage [37], was increased as well. Concordant with previous studies [7, 38], we established the experimental model of injured kidney induced by endotoxic shock by systemic LPS exposure.

To our knowledge, the application time and frequency of electroacupuncture stimulation seems critical for producing a prophylactic effect [39]. Hence, acupuncture was delivered 5 days before LPS injection with the disperse-dense wave (2Hz or 15Hz) and the intensity of less than 1.0 mA to exert renoprotection effects in current study [21, 40]. From another aspect, HO-1 with the subsequent metabolites of heme catabolism possessed intriguing signaling properties affecting numerous critical cellular functions, including inflammation, oxidative stress, cellular proliferation, and cellular survival [8]. Data from this study verified the direct involvement of HO-1 in mitigating LPS-induced acute kidney injury by electroacupuncture treatment at ST36 and PC6 acupoints.

In response to oxidative stress or inflammatory, Nrf2, as a bZIP transcription factor, dissociated from its cytosolic inhibitor, kelch-like ECH-associated protein (Keap1), and translocated to the nucleus, dimerized with a cis-element referred to as antioxidant response element (ARE) and ultimately transcribed its target genes [41]. Activation of Nrf2/ARE pathway has been reported to account for the therapeutic effects of HO-1 induction against sepsis [42]. Besides, as a conserved family of signal transduction, phosphoinositide 3-kinases (PI3K) are involved in nuclear translocation of Nrf2 via actin cytoskeletal changes [43]. Kuwana H et al. indicated [44], PI3K/Akt pathway lessened cisplatin-induced acute kidney injury and played a critical role in the maintenance of renal function. Consequently, upregulated of phospho-Akt protein, HO-1 protein, Nrf2 total and nucleoprotein expressions by electroacupuncture treatment was displayed in renal tissue, followed with the increments of Nrf2 protein accumulated in the nucleus by immunofluorescence staining. In contrast, wortmannin, the selective PI3K inhibitor, moderately eliminated above beneficial effects of electroacupuncture, indicating the protection against acute kidney injury during endotoxic shock was mediated through PI3K/Akt/Nrf2 pathway.

There were some limitations to this study that we would like to acknowledge. Firstly, Coincident with previous studies [45], both the levels of Nrf2 total protein and nucleoprotein were significantly upregulated by electroacupuncture treatment, which called for further exploration. Secondly, urine NAG excretion was used as a marker of renal tubular damage in current study, however, needed to be further evaluated in clinical settings [46]. Hence, it is desirable to develop novel biomarkers for early diagnosis of acute kidney injury, especially in septic patients. Finally, wortmannin pretreatment made a little difference to the expressions of HO-1, Nrf2 nucleoprotein and total protein, which were not in consistent with levels of p-Akt protein. The contradictory results indicated that PI3K/Akt may be not the only pathway responsible for electroacupuncture-afforded protection against LPS-induced AKI. Besides, the P38 MAPK, ERK, and PKC signaling pathways have been reported to mediate the phosphorylation of Nrf2 and the expression of HO-1 [47]. Therefore, to identify which signal cascade facilitated the activation of Nrf2/ARE by electroacupuncture and the interplay between these cascades and PI3K/Akt pathway still required further investigation.

Based on the data from current study, we concluded that treatment with electroacupuncture at ST36 and PC6 acupoints could ameliorate acute kidney injury induced by LPS in rabbits, the underlying mechanism of which involved the upregulation of HO-1 through the modulation of PI3K/Akt/Nrf2 pathway. This present finding aspires to lay a foundation for potential future clinical applications of electroacupuncture for acute kidney injury induced by endotoxic shock.

## Supporting Information

S1 Checklist The ARRIVE Guidelines Checklist. Animal Research: Reporting In Vivo Experiments.
(DOC)
