# Supplementary material for: Electroacupuncture Ameliorates Acute Renal Injury in Lipopolysaccharide-Stimulated Rabbits via Induction of HO-1 through the PI3K/Akt/Nrf2 Pathways
Source: PLoS One. 2015 Nov 2;10(11):e0141622. doi: 10.1371/journal.pone.0141622 (PMC4629879; doi:10.1371/journal.pone.0141622)

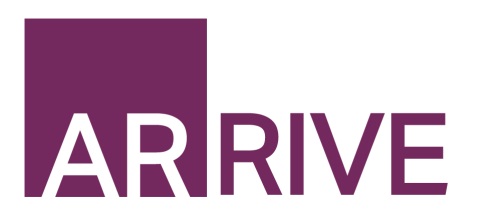


The ARRIVE Guidelines Checklist

Animal Research: Reporting In Vivo Experiments

Carol Kilkenny^1^, William J Browne^2^, Innes C Cuthill^3^, Michael Emerson^4^ and Douglas G Altman^5^

*^1^The National Centre for the Replacement, Refinement and Reduction of Animals in Research, London, UK, ^2^School of Veterinary Science, University of Bristol, Bristol, UK, ^3^School of Biological Sciences, University of Bristol, Bristol, UK, ^4^National Heart and Lung Institute, Imperial College London, UK, ^5^Centre for Statistics in Medicine, University of Oxford, Oxford, UK.*

|  | | ITEM | RECOMMENDATION | Section/ Paragraph |
| --- | --- | --- | --- | --- |
|  | 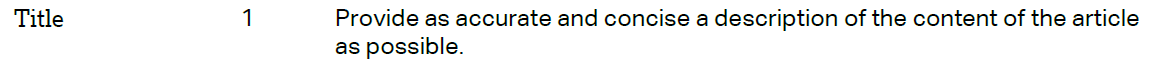 | | | Title |
|  | 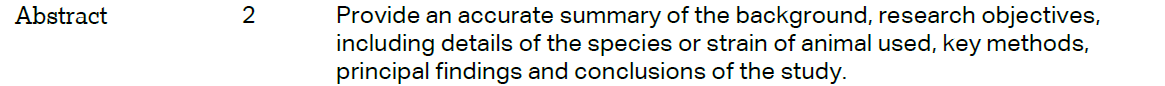 | | | Abstract |
|  | INTRODUCTION | | |  |
|  | 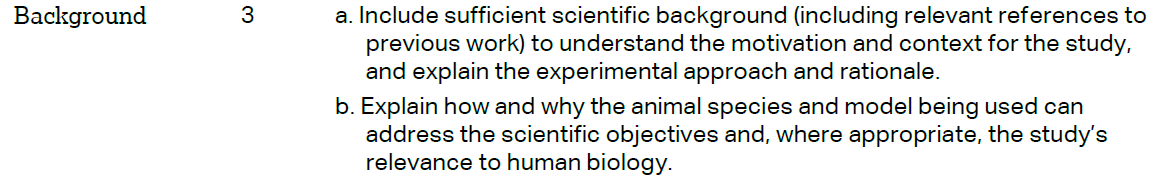 | | | Introduction,  Paragraphs  1-4  Paragraphs  1-3 |
|  | 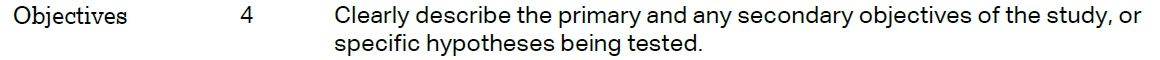 | | | Introduction,  Paragraph 4 |
|  | METHODS | | |  |
|  | 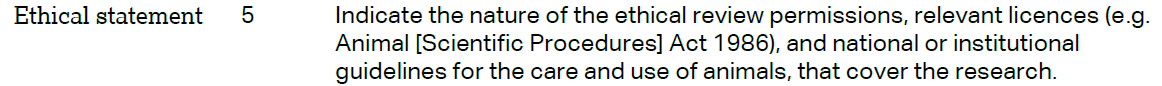 | | | Methods,  Paragraph 1 |
|  | 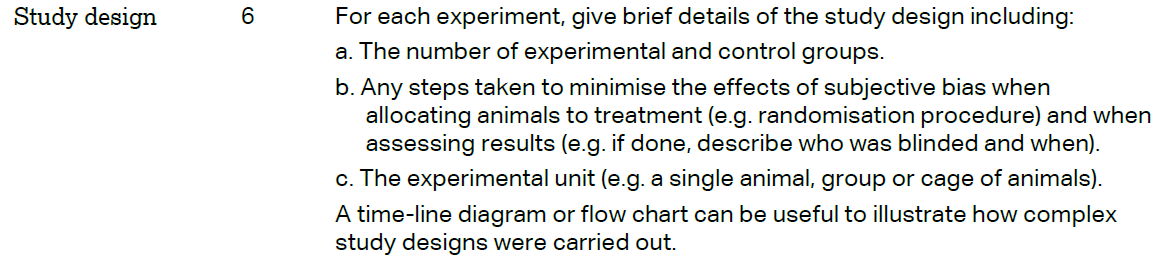 | | | Methods,  Paragraphs  3-4  Paragraph10  Paragraph 4  Figure 1 |
|  | 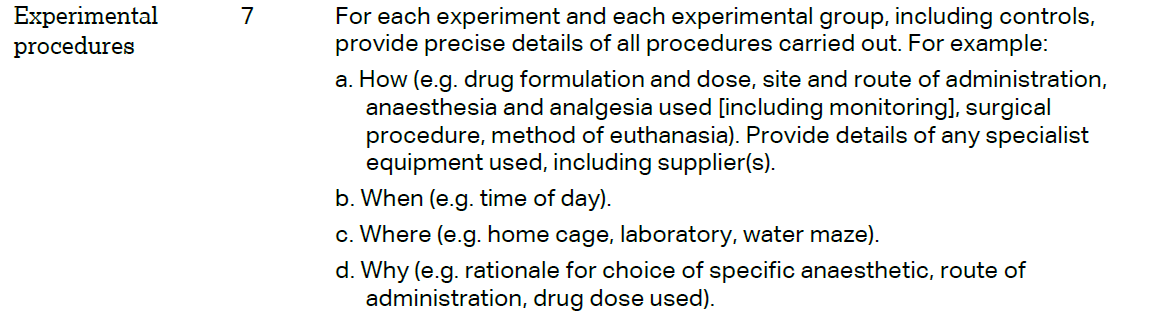 | | | Methods,  Paragraphs  2-9 |
|  | 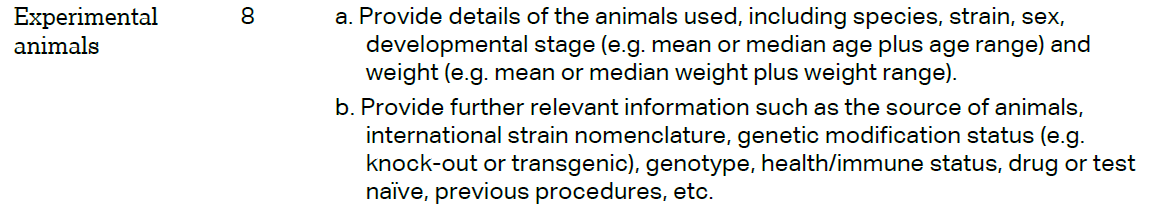 | | | Methods,  Paragraph 1 |

The ARRIVE guidelines. Originally published in *PLoS Biology*, June 2010^1^

|  | 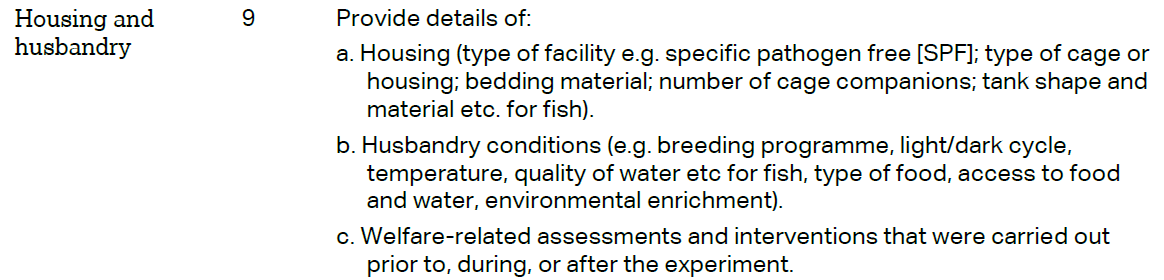 | Methods,  Paragraph1 | |
| --- | --- | --- | --- |
|  | 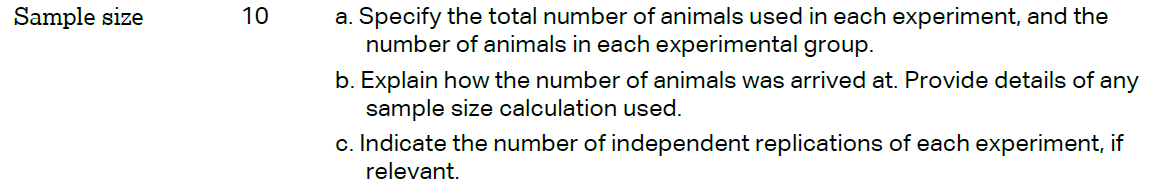 | Methods,  Paragraphs  1,4&10 | |
|  | 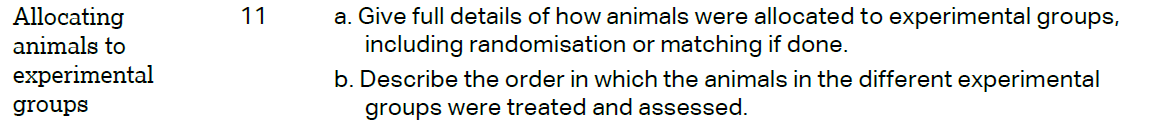 | Methods,  Paragraphs  2-5 | |
|  | 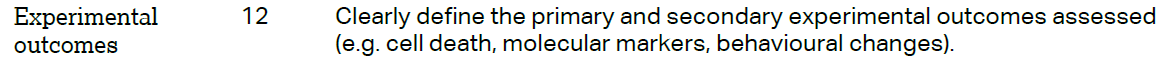 | Methods,  Paragraphs  5-9 | |
|  | 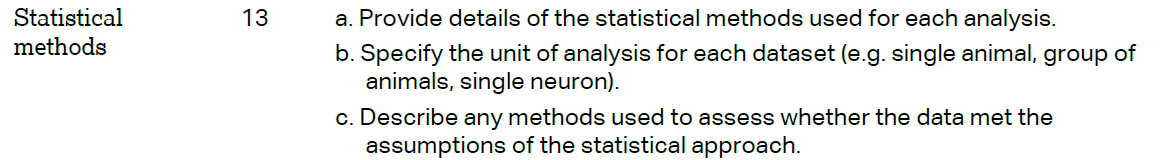 | Methods,  Paragraph 10 | |
|  | RESULTS |  | |
|  | 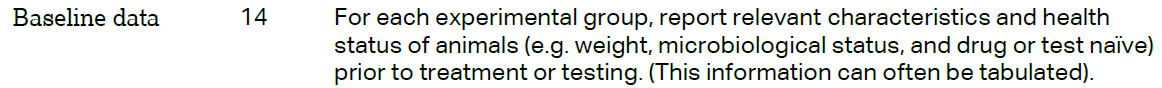 | Methods,  Paragraph 1 | |
|  | 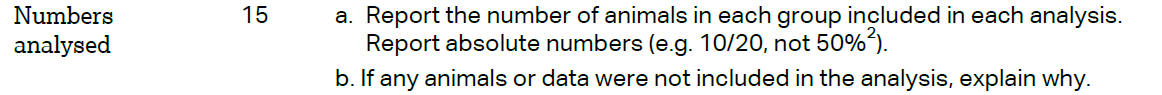 | Methods,  Paragraph 4 | |
|  | 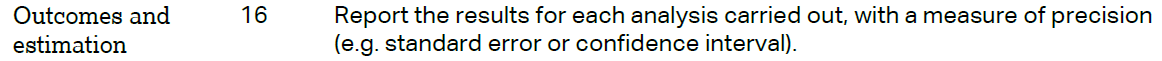 | Results,  Paragraph1-6 and Figure 2-5 | |
|  | 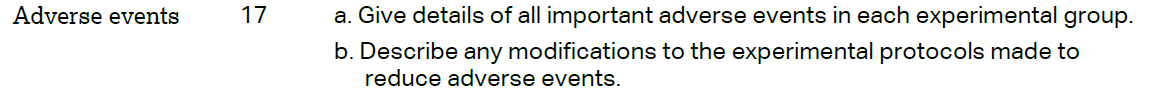 | Results, Paragraphs  1-6 | |
|  | DISCUSSION |  | |
|  | 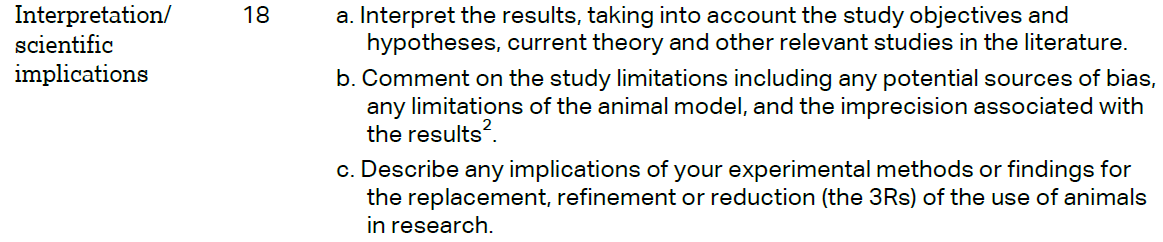 | Throughout  Discussion,  Paragraph 5  Paragraphs  2-4 | |
|  | 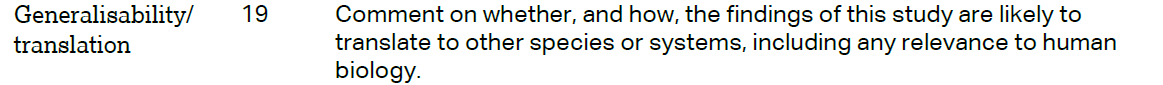 | Discussion,  Paragraphs  1&6 | |
| 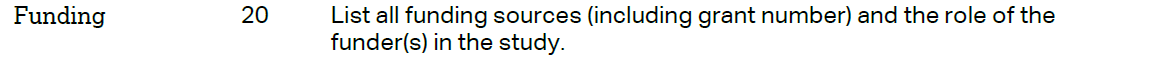 | | Line 8-10 of Page 1 |  |


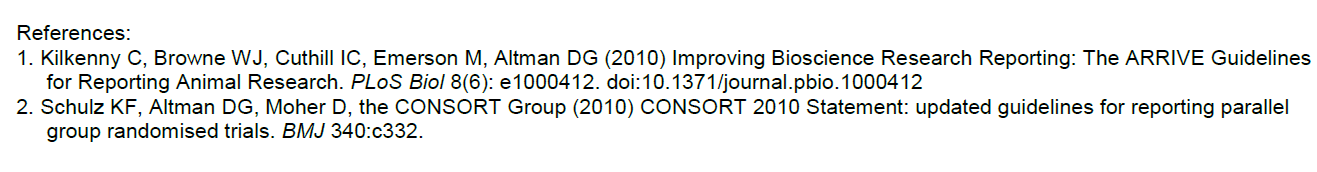

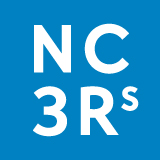

Supplement: S1 Checklist — Animal Research: Reporting In Vivo Experiments. (DOC) [file pone.0141622.s001.doc]
